# *Phyllanthus emblica* Fruit Extract Activates Spindle Assembly Checkpoint, Prevents Mitotic Aberrations and Genomic Instability in Human Colon Epithelial NCM460 Cells

**DOI:** 10.3390/ijms17091437

**Published:** 2016-09-03

**Authors:** Xihan Guo, Xu Wang

**Affiliations:** 1 School of Life Sciences, Yunnan University, Kunming 650091, China; guoxihan@aliyun.com; 2 School of Life Sciences, The Engineering Research Center of Sustainable Development and Utilization of Biomass Energy, Yunnan Normal University, Kunming 650500, China

**Keywords:** *Phyllanthus emblica*, mitotic aberrations, genomic instability, spindle assembly checkpoint, *BubR1*

## Abstract

The fruit of *Phyllanthus emblica* Linn. (PE) has been widely consumed as a functional food and folk medicine in Southeast Asia due to its remarkable nutritional and pharmacological effects. Previous research showed PE delays mitotic progress and increases genomic instability (GIN) in human colorectal cancer cells. This study aimed to investigate the similar effects of PE by the biomarkers related to spindle assembly checkpoint (SAC), mitotic aberrations and GIN in human NCM460 normal colon epithelial cells. Cells were treated with PE and harvested differently according to the biomarkers observed. Frequencies of micronuclei (MN), nucleoplasmic bridge (NPB) and nuclear bud (NB) in cytokinesis-block micronucleus assay were used as indicators of GIN. Mitotic aberrations were assessed by the biomarkers of chromosome misalignment, multipolar division, chromosome lagging and chromatin bridge. SAC activity was determined by anaphase-to- metaphase ratio (AMR) and the expression of core SAC gene budding uninhibited by benzimidazoles related 1 (*BubR1*). Compared with the control, PE-treated cells showed (1) decreased incidences of MN, NPB and NB (*p* < 0.01); (2) decreased frequencies of all mitotic aberration biomarkers (*p* < 0.01); and (3) decreased AMR (*p* < 0.01) and increased *BubR1* expression (*p* < 0.001). The results revealed PE has the potential to protect human normal colon epithelial cells from mitotic and genomic damages partially by enhancing the function of SAC.

## 1. Introduction

Medicinal plants have been used as a resource of remedies for the treatment of various diseases since ancient times [1]. They still play a significant role in primary health care in many developing countries and have attracted renewed interest in developed countries over the last decades. Moreover, since natural products are an important source of drugs in medicine, the interest in medicinal plants has increased together with the number of investigations into their biological effects on humans [2]. Recently, various studies showed that extracts from medicinal plants possess pharmacological activities including antioxidative, antigenotoxic and anticancer effects [3,4], as well as possess sub-acute and chronic toxicity including carcinogenic, genotoxic, and teratogenic effects [5]. These studies demonstrate that both the scientific validation of the ethno-medicinal claims and the safety evaluation are important for the expanding use of herbal plants in modern medicines.

*Phyllanthus emblica* Linn. (PE, syn. *Emblica officinalls* Gaertn.) of Euphorbiaceae family, commonly known as Yu-gan-zi in China, is a fruited plant distributed in tropical and subtropical areas of China, India, Thailand, Indonesia and the Malay Peninsula. The fruit of PE is an important dietary source of Vitamin C, minerals, and amino acids [6]. Entire parts of the plant, particularly the fruit, have been extensively used as a folk medicine by many folk systems, including traditional India medicine (Ayurveda), traditional Chinese medicine and Arab medicine (Unani) [1,6]. The regular consumption of PE is considered to be extremely useful in enhancing digestion, reducing constipation, reducing fever, purifying blood, reducing cough, alleviating asthma, strengthening the heart, benefiting the eyes, stimulating hair growth, enlivening the body, enhancing intellect, losing weight, delaying aging and extending healthspan [1,6]. Previous studies have demonstrated that PE possesses antioxidant, anti-mutagenic, anti-diabetic and anti-inflammatory properties, and protection for multiple organs, including brain, heart, liver, kidney, and stomach [6,7,8,9]. Thus, PE has been considered as a functional food due to the predominant medicinal functions beyond its adequate nutritional effects [10].

Genomic instability (GIN), a predominant hallmark of cancer, refers to the accumulation or acquisition of numerical and/or structural abnormalities in chromosomes [11]. Mechanisms leading to GIN are diverse and incompletely understood. The primary mechanisms causing GIN arise from mitotic aberration [12]. For example, defects in kinetochore-microtubule attachment can lead to GIN by promoting chromosome misalignment (CMA) at metaphase [13]. Deregulation of spindle assembly checkpoint (SAC), an essential self-monitoring system that ensures equal chromosome segregation, results in chromosome lagging (CL) and chromatin bridge (CB) during ana-telophase [14]. GIN can also arise from multinucleated/polyploid cells that usually divide in a multipolar manner due to supernumerary centrosomes and increased chromosome numbers [15].

Previously, we found the fruit extract of PE could kill colorectal cancer cells by elevating GIN in them [8]. This raises an important and intriguing question concerning whether PE can also induce GIN in normal colon epithelial cells. The present study aimed to determine the effects of PE on mitosis fidelity and genomic integrity of normal NCM460 colonic epithelial cells. To this end, frequencies of micronuclei (MN), nucleoplasmic bridge (NPB) and nuclear bud (NB) in cytokinesis-block micronucleus assay were used as indicators of GIN. Mitotic aberration was assessed by the biomarkers of CMA, multipolar division, CL and CB. The activity of SAC was determined by anaphase-to-metaphase ratio (AMR) and the expression of core SAC gene *BubR1*.

## 2. Results

### 2.1. Phyllanthus emblica Linn. (PE) Shows No Cytotoxicity

**Figure 1:**
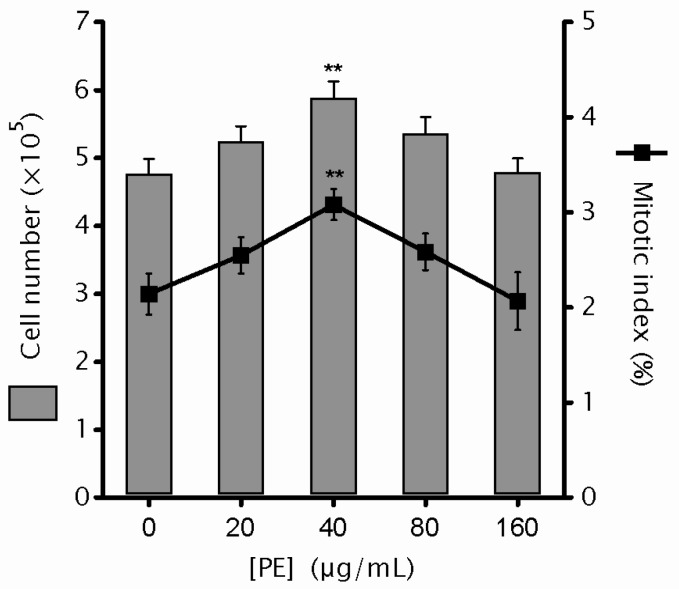
Influence of *P. emblica* (PE) treatment on cell growth rate of NCM460 cells. NCM460 cells with a seeding density of 1 × 10^5^/mL were incubated without or with PE (20–160 µg/mL) for 72 h, then cells were harvested and scored for total cell numbers (gray bars) and mitotic index (line with black squares). Total cell numbers were determined by counting live cells on a hemacytometer with the inclusion of trypan blue. Mitotic cells were scored based on their cell shape and DNA morphology after cells were fixed and stained with Giemsa. Five separate fields with >100 cells each were counted. Data are expressed as mean values ± SEM. ** *p* < 0.01 vs. untreated control.

Previously, we found that the 72-h treatment of PE at the doses ranging from 20 to 160 µg/mL have significant cytotoxicity to Colo320 colorectal cancer cells [8]. To test the cytotoxicity to human normal colon cells at the same dose range, NCM460 cells were cultured in medium containing 0, 20, 40, 80 and 160 µg/mL PE for 72 h. The data showed that, when compared to control, a steady increase in cell number was observed in 20–40 µg/mL PE cultures and the statistical significance was achieved at 40 µg/mL PE (*p* = 0.004). However, a steady decline in cell number was observed with PE dose further elevated when compared to that of 40 µg/mL PE, and the cell number at 160 µg/mL PE was comparable to that from the control (Figure 1). Moreover, cells were harvested and the mitotic index, indicative of cell growth, was assayed. Consistently, a similar bell-shaped dose response for PE was found in mitotic index (Figure 1). These results revealed that no cytotoxicity to NCM460 cells was associated with PE treatment. Coupled with our previous results [8], PE shows selective cytotoxicity to colorectal cancer cells, while leaving their normal counterparts undamaged.

### 2.2. PE Decreases the Rate of Genomic Instability (GIN)

**Figure 2:**
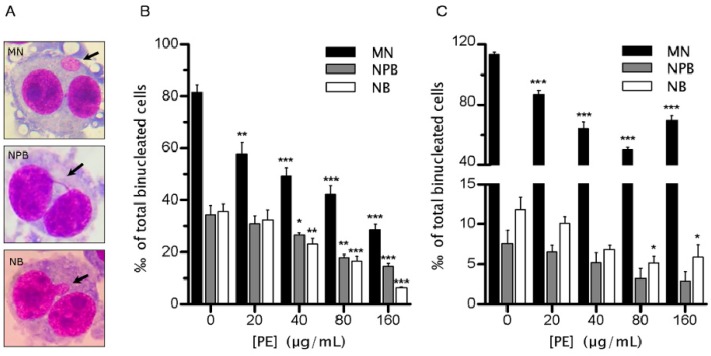
*P. emblica* (PE) treatment decreased the rate of endogenous genomic instability (GIN) in NCM460 cells. NCM460 cells were treated with PE (20–160 µg/mL) for 72 h, then their endogenous GIN was determined by cytokinesis-block micronucleus assay. (**A**) Representative images of the micronucleus (MN), nucleoplasmic bridge (NPB) and nuclear bud (NB) in binucleated cells (BNC) (indicated by arrows; 100× oil objective, Giemsa staining). Giemsa-stained DNA is in red, and the cytoplasm is in blue; (**B**,**C**) Frequencies of MN (black bars), NPB (gray bars) and NB (open bars) in control and cells treated with indicated doses of PE were determined in cytochalasin B-induced BNC (**B**) and spontaneous BNC (**C**). Data represent the mean of three independent experiments and SEM. * *p* < 0.05; ** *p* < 0.01; *** *p* < 0.001 vs. untreated control.

Previously, we found PE elevates the GIN rate of Colo320 cells to a catastrophic level to kill them [8], raising an important and intriguing question concerning whether PE can also induce GIN in normal colon epithelial cells. To investigate the effect of PE on the rate of GIN in NCM460 cells, we used the well-estimated CBMN assay, in which the biomarkers of MN, NPB and NB (Figure 2A) in cytochalasin B-induced binucleated cells (BNC) provide a reliable measure of GIN at both the single-cell level and the entire-population level [16]. Over the concentration range examined, MN, NPB and NB frequencies in cytochalasin B-induced BNC decreased as the concentration of PE was increased (Figure 2B). The effect of PE on decreasing MN frequency was the most pronounced, with the lowest tested dose (20 µg/mL) causing a significant decrease (*p* < 0.01) in MN frequency, and a 64.59% decrease in MN frequency was observed at the dose of 160 µg/mL. As for NPB and NB reduction, they also followed a dose-dependent trend with a significant decrease after 40, 80 and 160 µg/mL treatment (*p* < 0.05). Of note, PE induced a 57.57% and 81.86% reduction of NPB and NB frequencies, respectively, at the dose of 160 µg/mL (Figure 2B).

Besides in cytochalasin B-induced BNC, MN, NPB and NB frequencies in spontaneous BNC also decreased as the concentration of PE increased (Figure 2C). The inhibition of MN was evident from 20 µg/mL PE (*p* < 0.001) and reached a maximum at 80 µg/mL (reduced 56.1%). However, the inhibition tended to be weakened at 160 µg/mL. PE had potential to decrease the rate of NPB, although no significance was achieved. The decrease of NB by PE was only evident at doses of 80 and 160 µg/mL. Together, these data demonstrated PE possessed remarkable potential to decrease the rate of spontaneous GIN in NCM460 cells.

### 2.3. PE Prevents Defects in Chromosome Alignment and Segregation

**Figure 3:**
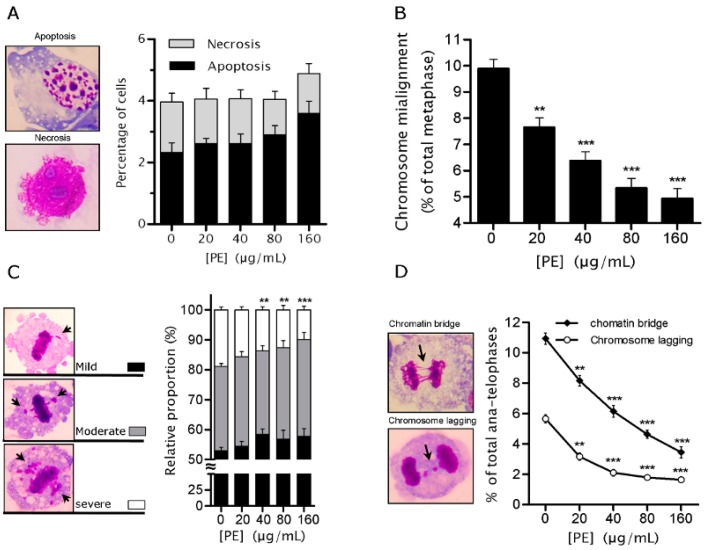
*P. emblica* (PE) treatment prevented mitotic aberrations in NCM460 cells. NCM460 cells were treated with PE (20–160 µg/mL) for 72 h, and then cells were fixed and stained to quantify assessment of cell death and mitotic aberrations. (**A**) Representative images show apoptosis and necrosis (**left panel**; 100× oil objective, Giemsa staining) and their incidences in indicated PE doses were quantified (**right panel**); (**B**) Quantification of chromosome misalignment in NCM460 cells from indicated PE doses. A minimum of 200 metaphases was scored for each dose in each of at least three independent experiments; (**C**) For analysis, chromosome misalignment was assessed with mild (one misaligned chromosome), moderate (2–5) and severe (>5) (identified by arrows in left panel; 100× oil objective, Giemsa staining), and their incidences in indicated PE doses were quantified (**right panel**); (**D**) Representative images show the chromatin bridge, chromosome lagging during ana-telophase (denoted by arrows in the left panel; 100× oil objective, Giemsa staining) and their incidences in indicated PE doses were quantified (**right panel**). A minimum of 50 metaphases was scored for each dose in each of 14 independent experiments. Giemsa-stained DNA is in red, and the cytoplasm is in blue. Data are expressed as mean values ± SEM. ** *p* < 0.01, *** *p* < 0.001 vs. untreated control.

To uncover the mechanisms underlying the decreased GIN, we determined the frequencies of apoptosis and necrosis because they can mimic GIN prevention by interfering with the appearance and survival of GIN cells. The results showed that PE had no significant potential to induce cell death (*p* > 0.05; Figure 3A). As shown in Figure 3A, although apoptosis frequency tended to elevate as the concentration of PE increased (*p* > 0.05), necrosis rate decreased (*p* > 0.05). These data confirmed that PE showed no obvious cytotoxicity to NCM460 cells under the tested dose range, and also indicated that the decreased GIN was not due to the elimination of GIN cells by cell death.

To investigate whether the decreased GIN was mediated by protecting against mitotic aberrations, we examined the chromosomal behaviors in fixed mitotic figures. The results showed that PE were able to significantly decrease CMA in a dose-dependent manner (Figure 3A). Notably, the average frequency of CMA was decreased by 50.11% in 160 µg/mL PE as compared to control. Moreover, we found PE had potential to dramatically reduce (*p* < 0.001) the proportion of severely misaligned metaphase (>5 misaligned chromosomes per metaphase plate) (Figure 3B). We found PE could prevent CMA during ana-telophase. Cells treated with PE showed a dose-related decrease in the frequency of CL (*p* < 0.001; Figure 3C), with a 71.05% decrease in the highest tested dose (160 µg/mL). In addition, we observed that PE treatment resulted in a significant decrease in CB in a dose-dependent manner (*p* < 0.001; Figure 3C). PE at 160 µg/mL caused a 68.46% decrease of baseline CB. Together, our results suggested that PE might exert its role in protection against GIN partially by preventing defects in chromosome alignment and segregation.

### 2.4. PE Prevents Multinucleation and Multipolar Mitosis

**Figure 4:**
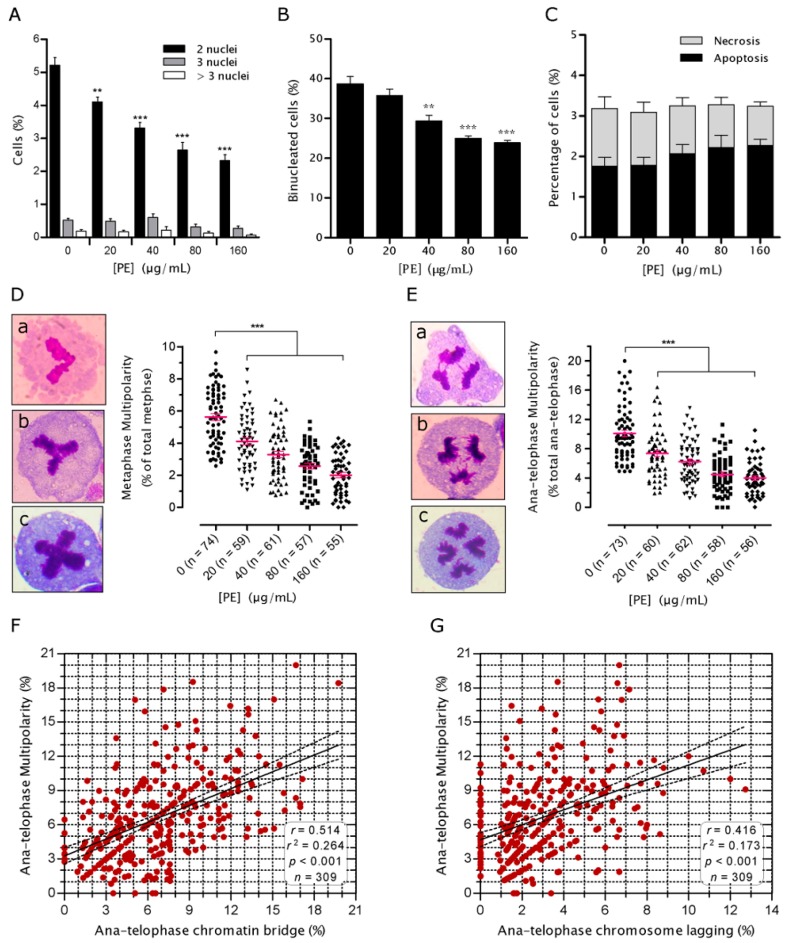
*P. emblica* (PE) treatment prevented NCM460 cells from multinucleation and multipolar mitosis. (**A**) Quantification of the multinucleation (≥2 nuclei) indices of in NCM460 cells from indicated PE doses. NCM460 cells were treated without or with PE (20–160 µg/mL) for 72 h, then cells were immediately fixed and stained for quantitative analysis of multinucleation and multipolarity; (**B**,**C**) Effects of PE on the frequency of binucleated cells (**B**) and cell death (**C**) in the presence of cytochalasin B. NCM460 cells were incubated for 24 h with the indicated doses of PE in combination with cytochalasin B (1.5 µg/mL). A minimum of 500 cells per PE dose from each of at least three independent experiments was analyzed; (**D**) Representative examples in left panel show the L-shaped (**a**), Y-shaped (**b**) and cross-shaped (**c**) arrangement of spindle poles (100× oil objective, Giemsa staining). Quantification of the percentage of multipolar metaphases from indicated PE doses is shown in right panel. Each mark depicts the quantification of one case from >200 metaphases; (**E**) Representative images in left panel show the tri-polar (**a**,**b**) and tetra-polar (**c**) anaphases (100× oil objective, Giemsa staining). The right panel shows the quantification of ana-telophase multipolarity from indicated PE doses. Each mark depicts the quantification of one case from >50 ana-telophases. In (**D**,**E**), Giemsa-stained DNA is in red, and the cytoplasm is in blue; *n* indicates the total cases quantified per PE dose; horizontal lines indicate the mean values and error bars depict the SEM (**F**,**G**). Linear regression plot showing the correlations between chromatin bridge (CB; **F**), chromosome lagging (CL; **G**) and ana-telophase multipolarity. Each red spot is representative of one pair of multipolarity and CB or CL from an individual case. The solid line is the weighted regression and the dotted lines are 95% confidence bands. *r*, Pearson’s correlation; *n*, the total quantified pairs. Data are expressed as mean values ± SEM. ** *p* < 0.01; *** *p* < 0.001 vs. untreated control.

Multinucleated cells (MNC), typically resulting from cytokinesis failure, have been proposed as a transient intermediate that lead to mitotic aberrations in daughter cells [15]. This notion led us to investigate whether PE has ability to prevent NCM460 cells from multinucleation. PE treatment for 72 h led to a dose-dependent decrease in the proportion of BNC (*p* < 0.001; Figure 4A). Moreover, the spontaneous tri- and tetra-nucleated cells also tended to be decreased when PE doses increased, although no significance was achieved either (Figure 4A). At the highest dose of PE, a 72-h treatment reduced the incidence of MNC to 44.45% of that in untreated control.

Since chromatin trapped in the cleavage plane, such as CB and CL, is a main cause of spontaneous cytokinesis failure [17], we expected the decreased ratio of MNC might be partially due to the decreased CB and CL. Indeed, regression analysis revealed significant correlations between CB (Pearson *r* = 0.576, *p* < 0.001), CL (Pearson *r* = 0.265, *p* = 0.048) and MNC. However, since most of the spontaneous BNC were lacking CB and CL (indicated by NPB and MN; Figure 2C), we next wondered whether PE would be capable of impeding the de novo formation of BNC. We found that the BNC-inducing effect of cytochalasin B, a drug that blocks the assembly of contractile actomyosin ring, was significantly reduced in the presence of PE (*p* < 0.001; Figure 4B), which did not concomitantly increase the frequency of cell death (*p* > 0.05; Figure 4C ). These results suggested that PE reduced the generation of spontaneous BNC which could also result from the prevention of defects in cytokinetic apparatus.

When MNC undergo mitosis, the inherited amplified centrosome can lead some of them to multipolar mitosis [15,18]. Thus, we set out to assess whether PE has potential to decrease the frequency of multipolar mitosis. Multipolar mitosis can be readily distinguished by routine staining at metaphase through telophase [15,19]. Interestingly, a relatively high proportion of metaphases (5.6%) and ana-telophases (10%) showed configurations obviously originating from more than two spindle poles. Further analysis of mitotic cells showed that PE treatment, even in its lowest tested dose (20 µg/mL), was able to significantly decrease (*p* < 0.001) the frequencies of multipolar metaphase (Figure 4D) and multipolar anaphase (Figure 4E). In the highest tested dose, there was a 64.47% and 59.85% decrease in metaphase and ana-telophase multipolarity as compared to the corresponding control, respectively. Moreover, strong correlations were found between CB (Figure 4F), CL (Figure 4G) and ana-telophase multipolarity. This is in accordance with the notion that CB and mitotic multipolarity may be correlated because one of the phenomenon is directly dependent on the other [20,21]. Together, these results suggested that PE might also exert its role in protection against GIN by preventing the generation and division of genetically unstable MNC.

### 2.5. PE Activates the Spindle Assembly Checkpoint (SAC)

**Figure 5:**
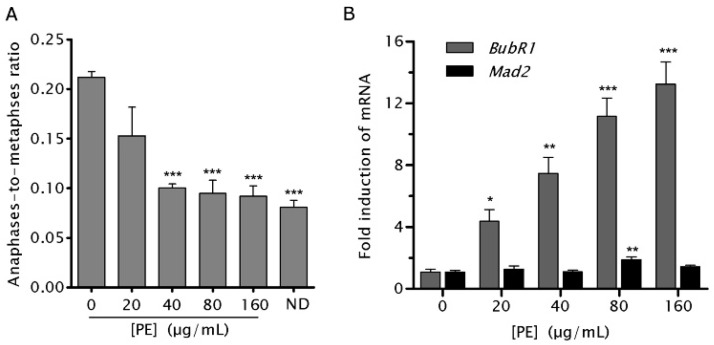
*P. emblica* (PE) treatment activated the spindle assembly checkpoint (SAC) and upregulated the expression of budding uninhibited by benzimidazoles related 1 (*BubR1*) in NCM460 cells. (**A**) NCM460 cells were treated with indicated doses of PE or 300 ng/mL nocodozale (ND, positive control) for 4 h followed by recovery for 1 h before cells were harvested for determining anaphase-to-metaphase ratio (AMR), an estimated measurement of SAC activity. AMR was obtained by dividing the total number of anaphase cells by the total number of metaphase cells counted for each PE concentration; (**B**) NCM460 cells were treated with indicated doses of PE for 72 h, *BubR1* (gray bars) and mitotic arrest deficient 2 (*Mad2*; black bars) mRNA levels were measured by real-time quantitative polymerase chain reaction (PCR) after mRNA extraction and reserve transcription. Samples for each experimental group were run in triplicates and normalized to glyceraldehyde-3-phosphate dehydrogenase (*GAPDH*) mRNA levels. Results are expressed as fold increase in treated cells vs. untreated control. Values represent the means ± SEM of the fold induction taken from three independent experiments. * *p* < 0.05; ** *p* < 0.01; *** *p* < 0.001 vs. untreated control.

SAC is an essential self-monitoring system that inhibits anaphase onset in response to CMA [14]. The high percentage of NCM460 cells entering anaphase with CL, CB and multipolarity, coupled with their significant reduction after PE treatment, suggested NCM460 cells have a weakened SAC and which can be activated by PE. To test it, NCM460 cells were treated with PE for 4 h followed by recovery without PE for 1 h before cells were harvested for determining the AMR, an initially estimated method of examining the status of the of SAC [22]. A dose-dependent decrease (*p* < 0.01) in AMR was found in NCM460 cells treated with increasing concentrations of PE (Figure 5A). AMR in cells treated by 160 µg/mL was comparable to that from nocodazole (ND), a drug inducing robust SAC activity by disrupting the kinetochore-microtubule attachment at most chromosomes [14]. These results demonstrated that PE could prevent aberrant metaphases from entering anaphase prematurely through activating SAC.

To characterize how PE activated the weakened SAC, we examined the mRNA expression of two core SAC genes, *BubR1* and mitotic arrest deficient 2 (*Mad2*). We detected a significantly increased expression of *BubR1* in PE-treated compared with control NCM460 cells. However, quantitative polymerase chain reaction (PCR) analysis revealed that *Mad2* mRNA levels are only modestly increased in PE-treated compared with control NCM460 cells (Figure 5B). These data indicated that increased *BubR1* expression may be responsible for the activated SAC after PE treatment.

### 2.6. PE Enhances the Function of SAC

**Figure 6:**
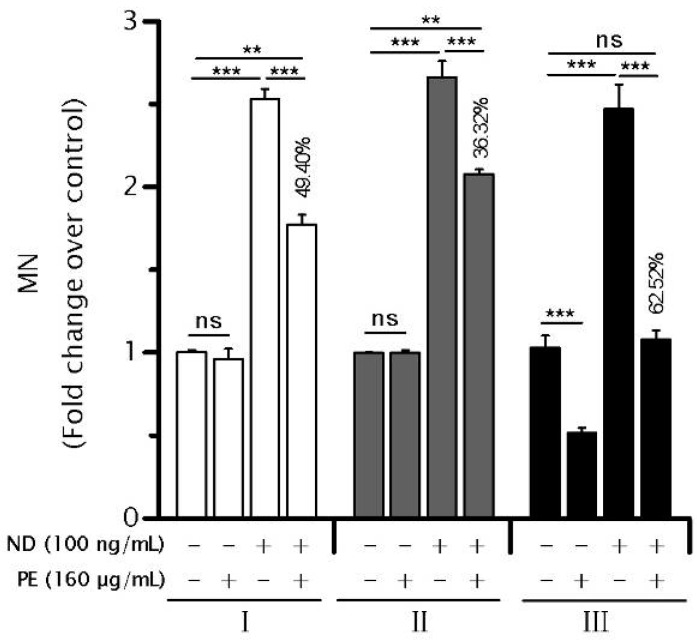
*P. emblica* (PE) treatment protected against nocodazole-induced micronuclei (MN) in NCM460 cells. NCM460 cells were either co-treated with PE and nocodazole (ND) for 6 h (I) or pre-treated with PE for 6 h (II) and 72 h (III) before a 6-h ND challenge. Cells were then allowed to recover for 1 h before cytochalasin B was added to block cytokinesis (10 h) for MN assessment. MN assessment obtained from more than 1000 binucleated cells and then were set relative to the values measured in untreated control cells, which were arbitrarily set at 1. Similar results were observed in three independent experiments. The numbers above bars indicate the percentages of reduction in ND-induced MN caused by PE from each experimental scheme calculated using the formula described in Materials and Methods. The symbols + and − represent cells were treated with or without indicated components, respectively. Error bars indicate one SEM. ** *p* < 0.01; *** *p* < 0.001 vs. untreated control. ns, not significant.

Although PE was found to activate the SAC, whether the function of SAC was indeed enhanced by PE remained unclear. To this end, we determined the effects of PE on MN frequency induced by ND which perturbs the functional integrity of SAC and results in the generation of MN [14,23]. NCM460 showed a significantly increased MN in response to exposure to ND (Figure 6). Consistent with our prediction, we found that co-treating NCM460 cells with PE (160 µg/mL) can slightly, yet still significantly (*p* < 0.001) decrease ND-induced MN (Figure 6). Similarly, pre-treating NCM460 cells with PE for 6 h could yield a slight but significant reduction in ND-induced MN. Moreover, a longer PE pre-treatment (72 h) could decrease the ND-induced MN to a level comparable to that of untreated control (Figure 6). Together, these data indicated that PE treatment could enhance the function of SAC in NCM460 cells.

## 3. Discussion

Studies during the past two decades have shown that PE can modify GIN induced by various exogenous carcinogens and mutagens (reviewed in [6]). However, studies about its effect on spontaneous GIN are still lacking. We defined here, for the first time, to our knowledge, that PE treatment significantly decreases the spontaneous GIN in NCM460. To explore the underlying mechanisms, we found PE has a dominant capacity in activating the impaired SAC in NCM460 cells through increasing *BubR1* expression, thereafter preventing CMA, CL, CB and multipolar mitosis.

The significant decrease of MN in NCM460 cells treated with PE is interesting. The majority of the spontaneously raised MN in human cells directly result from mitotic aberrations, including CL and CMA [24]. Some others result indirectly from mitotic defects, for example, fragmented CB [24] or persisted CB [25]. Irrespective of how MN is generated, micronucleated cells usually harbor complex chromosomal alterations both in their MN and their primary nuclei. The majority of MN undergo irreversible nuclear envelope collapses in interphase [26] and defective and asynchronous DNA replication, resulting in DNA damage and often extensive pulverization of the chromosome in MN [23]. More importantly, fragmented chromatin in MN undergo complex rearrangements, some of which exhibit the defining characteristics of chromothripsis, a phenomenon often seen in several cancers [27]. After mitosis, these mosaic chromosomes resulting from chromothripsis can be reincorporated into daughter nuclei, potentially integrating mutations from the MN to the genome [27]. If the MN-bearing cells enter the next mitosis, they will more frequently produce cells with additional MN [28], indicating the long-term impact of MN in promoting GIN in the population scale.

Another striking finding in the present study is that PE decreased the rate of CB in NCM460 cells. CB can be a consequence of dicentric chromosomes that either formed through interstitial chromosome rearrangements or because of defects in telomere structure or length [21]. CB has long been recognized as a resource of breakage-fusion-bridging cycle [25] and, more recently, it has been found as a source of chromothripsis [29]. Moreover, the chromatin in CB that localized to the cleavage plane can cause spontaneous furrow regression, resulting in the spontaneous generation of BNC in tissue culture cells [17]. BNC/tetraploid cells are frequently found in pre-neoplastic lesions and have been proposed as a genetically unstable intermediate, which can generate offsprings harboring massive GIN by two non-exclusive mechanisms: First, the inherited centrosome amplification in BNC/tetraploidy may lead them to enter multipolar mitosis. Because of the different levels of chromosomal capture and alignment on each pole, multipolar mitosis causes the near-to-stochastic segregation of chromosomes and hence produces massive GIN in most daughter cells [15,18,21]. Second, even when the supernumerary centrosomes are clustered to enable pseudo-bipolar mitosis, the possibility of merotelic kinetochore attachment is increased [30,31]. Merotelic attachment cannot be detected by SAC and the merotelically attached chromosome lags during anaphase and eventually is either lost or distributed into the wrong daughter cell to form MN [15,18].

Our results suggest that PE-induced decrease of spontaneous MNC may be a consequence of prevention of CB and CL, as well as the prevention of defects in cytokinetic apparatus. Although MNC is one factor that contributes to multipolar division [15,18], the frequency of spontaneous multipolarity in NCM460 cells was higher than that of MNC. Two plausible, but not mutually exclusive, mechanisms can be engaged to explain it: First, multipolar cells spend longer times in mitosis compared to their bipolar counterparts, not only because they have excessive chromosome numbers but also the presence of multiple microtubule asters may perturb the stability of kinetochore attachment [31]. Second, mitotic spindle multipolarity can be formed without centrosome amplification, instead as a consequence of misaligned chromosomes [32]. This processes occurs after spindle bipolarization, explaining the higher frequency of ana-telophase multipolarity than that of metaphase multipolarity in NCM460 cells. Therefore, the reduction of multipolarity by PE may be the consequence of preventing the generation and division of genetically unstable MNC, as well as the decrease of CMA.

According to our results, the prevention of mitotic defects and GIN by PE could be explained, at least in part, by the upregulation of *BubR1*. BubR1 is not only a central component of the SAC [14], but also a master regulator of GIN [33]. Dai et al. [34] found that *BubR1*^+/−^ mouse embryonic fibroblasts (MEF) exhibit an increased frequency of spontaneous formed MN compared with the *BubR1*^+/+^ MEF. Baker et al. [35] found that *BubR1*^H/H^ and *BubR1*^−/H^ MEF have a defective SAC and more ana-telophase figures with CL than in *BubR1*^+/+^ MEF. Wang et al. [36] found that *BubR1*^+/−^ MEF contained a large number of polyploid cells. Moreover, Lampson and Kapoor [37] found that depletion of *BubR1* causes severe CMA phenotype in HeLa cells. Izumi et al. [38] found that *BubR1* knockdown HeLa cells induce binucleation, centrosome amplification and multipolar division. Although the phenotype of *BubR1* in NCM460 cells remains to be determined, our finding that PE sustains a high *BubR1* level in NCM460 cells suggests PE can upregulate *BubR1* to prevent SAC defects, microtubule-kinetochore attachment defects, chromosome mis-segregation, as well as binucleation/polyploidization and mitotic multipolarity. This suggestion is supported by the findings that sustaining a high-level expression of *BubR1* preserves genomic integrity in MEF [39].

PE is a rich source of Vitamin C and many known medicinally relevant polyphenols such as gallic acid, ellagic acid, quercetin, geraniin, chebulagic acid, corilagin and phyllanthin [6,8,9,40]. This study was not aimed to characterize or isolate any active ingredients from PE and to look at its action. We think the effects of PE observed in the present study cannot be attributed to any single phytochemical, since it has been demonstrated that no single phytochemical can replace the combination of phytochemicals in PE in achieving its medicinal benefits [6]. Studies have shown that the effects of whole-plant extract cannot be mimicked by administering isolated and purified constituents of the herbs. Instead, it is now widely believed that the health benefits of the vast fruits and vegetables are attributed to the additive and synergistic effects of the complex mixture of phytochemicals present in them [41].

Much of effort has centered on the evaluation of PE′s potential in preventing GIN induced by exogenous chemicals and environmental pollutants (reviewed in [6]), while little attention is being paid to its potential in spontaneous GIN. This phenomenon is primarily owing to the fact that the frequency and ultimate importance of spontaneous GIN in normal tissues have not been fully appreciated [42]. The spontaneous GIN is defined as a definite level of background GIN that is normally registered in the cells after their division [43]. There is evidence for a high variation of spontaneous GIN frequency in human normal cells and this variation mainly depends on age, gender, race and susceptible genotypes. Recently, multiple studies have reported that ‘‘healthy’’ human somatic tissues may also be frequently affected by GIN, including point mutations, copy-number aberrations, retrotransposition, chromothripsis [42], aneuploidy [44] MN and NB [45,46]. In human lymphocytes, chromosomal mosaicism is detectable at a constantly low frequency of 0.23%–0.5% until the age of 50 years. Thereafter, however, this percentage rises rapidly to 2%–3% by the age of 75 years [47,48]. The age-related increase of spontaneous MN is gender dependent, with females having higher MN frequencies than males [45,46] while males have a higher frequency of chromosomal mosaicism than females [47]. A recent study indicates that relative to individuals of European and Asian ancestry, individuals of African ancestry are at a significantly reduced rate of chromosomal mosaicism in blood [49]. In addition, MN frequency in lymphocytes is positively associated with a family history of cancer [46], indicating susceptible phenotypes increase the spontaneous frequency of GIN. Recently, the number of cell divisions in a tissue is reported as an intrinsic factor for spontaneous GIN. Cells have a higher rate of turnover, for example, colorectal cells tend to accumulate a higher frequency of random mutations during DNA replication [50]. It has been estimated that the acquisition of GIN is intimately related to cancer [44,47,48]. GIN at the chromosomal level is a common feature of human tumors and occurs early in tumorigenesis [11]. Depending on the oncogenic and tumor suppressive effects of genes encoded by the instable chromosomes, individual GIN cell will have distinct proliferative and survival states. The vast majority of GIN events are non-viable, neutral or mildly deleterious, and some are tumor suppressive. However, some GIN events facilitate the origin and positive selection of advantageous karyotypes which activate and/or enhance multiple pathways that are integral to cell survival and proliferation [42]. Among these, catastrophic mutational processes such as chromothripsis induced by MN and CB may fuel a rapid accumulation of advantageous karyotypes, resulting in a transformation from normal to malignant cells [42]. It is likely that the role of GIN in diseases other than cancer is greater than documented and GIN may be an ultimate contributing factor to many diseases [51].

In our present study, we could find that PE can decrease the spontaneous GIN probably because a good in vitro cell model was used. NCM460 was derived from colon mucosal epithelial cells of a 68-year-old Caucasian male [52]. As mentioned above, these properties determine a high spontaneous GIN level in NCM460 cells. Moreover, instead of being immortalized by viral transformation, NCM460 was spontaneously immortalized [52]. This property makes NCM460 valuable in analysis of many cellular functions, in particular those related to genomic integrity, since virus-transformed cells are associated with spontaneous CIN that differ from their normal counterpart. Similar to the effects of PE, consuming fruits and leafy green vegetables is also found to reduce spontaneous MN frequency in human lymphocytes [45]. In addition, prolonged consumption (4 months) of a vitamin-antioxidant mixture (containing Vitamins A, C, E, as well as β-carotene, folic acid, and rutin) can significantly decrease spontaneous MN rate in lymphocytes from aged donors (63–82 years old), but not in lymphocytes from young donors (23–30 years old) [43]. Given that MN are accumulated in human cells with age [45,46], this study suggests micronutrients can protect against spontaneous MN in cells with a high background value. Together, this study suggests that PE has the potential to reduce spontaneous GIN in human cells and NCM460 may be used as a good in vitro cell model for identifying other fruits or micronutrients that possess the potential to prevent spontaneous GIN.

Numerous in vitro and in vivo studies have demonstrated that PE exert preventive effects against cancers, but the underlying molecular mechanisms remain elusive (reviewed in [6,7,9]). As we know, aging is the primary risk factor for cancer. The expression level of *BubR1* is decreased with age [35,39]. Mitotic accuracy and genomic integrity are largely affected when the levels of *BubR1* in the cell fall below a specific threshold concentration [35]. Compared with *BubR1*^+/+^ littermates, *BubR1*^+/−^ mice rapidly develop lung as well as intestinal adenocarcinomas in response to challenge with carcinogen [34]. However, sustained high-level expression of *BubR1* in aged transgenic mice preserves genomic integrity and reduces tumorigenesis, even in the presence of genetic alterations that strongly promote cancer [39]. Based on these results, it seems that pharmacological reinforcement of endogenous mechanisms against GIN offers a promising strategy for cancer prevention during aging. Our findings that PE elevates *BubR1* expression and prevents mitotic aberrations and GIN, thus, provide a molecular basis for cancer prevention by PE. The relationship between PE and *BubR1* upregulation, however, is complex, and further investigations could yield more insights into this process.

## 4. Materials and Methods

### 4.1. PE Extraction

The dry fruit of PE was provided by Yunnan Phytopharmaceutical Co., LTD (Kunming, China). Fifty grams of mashed PE was steeped in 500 mL of distilled water at room temperature for 2 h and then boiled for 10 min. This procedure was repeated three times to ensure maximum extraction. The supernatant was filtered and lyophilized. The yield of fruit was 5.66%. The major components of PE extract were l-ascorbic acid, gallic acid, corilagin, ellagic acid and phyllanthin [40]. A stock solution (5 mg/mL) of PE was prepared in RPMI 1640 medium (Gibco, New York, NY, USA) and sterilized by filtration. The stock solution was stored at −20 °C and thawed immediately before use.

### 4.2. Cell Culture and Treatment

The human-derived nontransformed colonic epithelial cells line NCM460 cell line [52] was obtained from INCELL (San Antonio, TX, USA) and maintained as a monolayer in RPMI 1640 medium (Gibco) supplemented with 10% newborn calf serum (Gibco), 1% penicillin (5000 IU/mL)/streptomycin (5 mg/mL) solution (Gibco), 1% l-glutamine (2 mM) (Sigma, St. Louis, MO, USA), and cultured at 37 °C and 5% CO_2_ in a humidified incubator. NCM460 cells were subcultured at a density of 1 × 10^5^/mL into 24-well plates or 6-well plates and cultured in RPMI1640 medium containing 0, 20, 40, 80 or 160 µg/mL PE for 72 h.

### 4.3. Cell Viability Analysis

After treatment, both rounded-up and attached cells were harvested by trypsinization and washed twice with phosphate buffer saline (PBS, pH 7.2). Cell viability was tested by trypan blue exclusion. Cell suspensions (5 µL) were stained with 5 µL trypan blue (Boster, Wuhan, China) and counted in a hemocytometer. This procedure was repeated three times.

### 4.4. Cytokinesis-Block Micronucleus (CBMN) Assay

After treatment, cells were washed with PBS (pH 7.2) twice and cultured in PE-free medium containing cytochalasin B (4.5 µg/mL; Sigma) for 24 h. Then, cells were detached from plates with 0.25% trypsin (Gibco) to generate a single-cell suspension. Cells were centrifuged onto glass slides with the final density kept between 0.5 × 10^5^ and 1 × 10^5^ cells per slide by cytospin (San’ersi, Shanghai, China) at 800 rpm for 5 min. Slides were fixed in 100% methanol at −20 °C for 15 min. Fixed cells were then stained with 5% Giemsa in PBS (pH 6.8). The slides were distained twice in double-distilled H_2_O, followed by air dry and cover slip. Before scoring, slides were stored protected from light. For determining the frequency of MN, NPB or NB, at least 1000 interphase BNC with well-separated nuclei were scored according to criteria previously described [53]. All CBMN slides were coded by another person not involved in this study and scored blind using the same light microscope (CX21, Olympus, Tokyo, Japan) to eliminate slide scoring bias.

### 4.5. Mitotic Index and Mitotic Aberration Analysis

After treatment, cells were detached, centrifuged to slides, fixed, stained and covered as described above. The mitotic cells which possessed condensed chromosomes were microscopically distinguished from the interphase cells. The mitotic index, defined as the percentage of mitotic cells in the total cell population, was determined by the number of mitotic cells per 500 cells.

CMA was defined as one or a few chromosomes scattered apart from the metaphase plate in the cytoplasm; CL as one or a few chromosomes lagged behind at the spindle equator while all other chromosomes moved toward the spindle poles during ana-telophase; CB as one or a few DNA fibers with the thickness of an entire arm connecting both anaphase chromosome packs during ana-telophase. For analysis, we assessed CMA based on its incidence with “mild” referring to only one misaligned chromosome in a metaphase plate, “moderate” between 2 and 5, and “severe” greater than 5. Multipolarity was analyzed at metaphase and ana-telophase. Metaphase multipolarity was scored when the metaphase plate exhibited either L-shaped, Y-shaped or cross-shaped rearrangement phenotype, as defined previously [19]. Ana-telophase multipolarity was scored in which the chromosomes were pulled towards 3 or more poles. A total of 200–450 metaphase cells were counted in each PE dose per experiment for CMA and metaphase multipolarity and 50–160 ana-telophases for CL, CB and ana-telophase multipolarity.

### 4.6. Evaluation of Cell Death and Multinucleated Cells (MNC)

The analysis of cell death (apoptosis and necrosis) and MNC was performed on the same slides prepared for mitotic aberrations analysis. As suggested by Fenech et al. [16,53], cells with chromatin condensation and intact cytoplasmic and nuclear boundaries as well as cells exhibiting nuclear fragmentation into smaller bodies within an intact cytoplasmic membrane were classified as apoptotic, while cells with cytoplasm loss, swelled nuclear and damaged nuclear membrane with only a partially intact nuclear structure and often with nuclear material leaking from the nuclear boundary were necrotic. Cells with ≥2 nuclei that were approximately equal in size were scored as MNC. To assess the effect of PE on cytochalasin B induced BNC, NCM460 cells were seeded in 24-well plates and incubated for 24 h with the PE (0, 20, 40, 80 and 160 µg/mL) in combination with cytochalasin B (1.5 µg/mL). Cells then harvested and stained for BNC and cell death analysis. Five hundred cells were counted for each sample. All these slides were coded by another person not involved in this study and scored blind using the same light microscope (CX21) to eliminate slide scoring bias.

### 4.7. Determination of SAC Activity

SAC activity was determined by the AMR as previously described [22]. NCM460 cells were treated with PE (0, 20, 40, 80 or 160 µg/mL) for 4 h. Then cells were washed twice with PBS and released into PE-free medium for 1 h before cell harvest, fixation and Giemsa staining. Nocodozale (300 ng/mL; Sigma), a microtubule poison that can produce an active SAC, was used as a positive control. AMR was obtained by dividing the total number of anaphase cells by the total number of metaphase cells. A minimum of 200 mitotic cells per PE dose from each of at least three independent experiments was analyzed.

### 4.8. Real-Time Quantitative Polymerase Chain Reaction (PCR)

After treatment, total RNA was isolated with high pure RNA isolation kit (Roche diagnostics, Indianapolis, IN, USA). cDNA was synthesized with PrimeScript RT reagent Kit with gDNA eraser (Takara, Japan) according to the manufacturer’s protocol. Real-time quantitative PCR (RT-qPCR) was performed in triplicates using the Kapa SYBR fast qPCR kit (KAPA Biosystems, Boston, MA, USA) and Applied Biosystems StepOne Plus RT-qPCR system (ABI, Foster City, CA, USA). The primer sequences for *BubR1* [54], *Mad2* [55] and *GAPDH* [56] were previously described. The samples were heated at 95 °C for 3 min followed by 40 cycles at 95 °C for 3 s and 60 °C for 30 s. Expression of *BubR1* and *Mad2* mRNA was normalized to expression of *GAPDH* in each sample and fold change was calculated using the 2^−ΔΔ*C*t^ method [57].

### 4.9. Micronucleus (MN) Induced by Nocodazole (ND)

NCM460 cells were treated as previously [23] with 100 ng/mL ND for 6 h to synchronize cells in mitosis. Nocodazole was then washed out and cells were released from the mitotic arrest for 1 h in fresh medium. Then, 3 µg/mL cytochalasin B was added to the medium and cells were incubated for 10 h to inhibit cytokinesis before cell harvest, fixation, and Giemsa staining.

To explore the impact of PE on nocodazole-induced MN, cells were simultaneously treated with PE (160 µg/mL) and nocodazole for 6 h, or pretreated with PE for 6 h or 72 h before a 6-h nocodazole treatment. The capacity of PE on reducing nocodazole-induced MN was calculated according to the formula adapted from [58]:

(1) $$\%R=\frac{A-B-(C-D)}{A-C}\times 100$$

where *%R* is the reduction percentage, *A* the MN frequency after treatment with ND, *B* the MN frequency after treatment with PE and ND, *C* the MN frequency of negative control, and *D* the MN frequency after treatment with PE.

### 4.10. Statistical Analysis

The Kolmogorov-Smirnov test was used to test the normality of all data sets, the results showed that all data sets were normally distributed (*p* > 0.05). The differences of observed values among the control and PE treated groups were analyzed using one-way analysis of variance (ANOVA). First, Levene′s test was performed to examine the homogeneity of variances among the control and PE treated groups. Post-hoc tests (Tukey’s test was used when the equality of variances assumption holds (*p* > 0.05), and the Dunnett’s *T3* test was used otherwise (*p* ≤ 0.05)) followed in case a significant effect (one-way ANOVA, *p* < 0.05) was detected. Two-tailed Pearson correlations were used to analyze relationships between two variables. We considered as significant only differences having a *p*-value (two-tailed) lower than 0.05. All statistical analyses were performed using SPSS 17.0 for windows (SPSS, Chicago, IL, USA).

## 5. Conclusions

In summary, this work is a novel attempt that explored the potential of PE in terms of mitotic fidelity and genomic integrity of human normal colon epithelial cells. Collectively, these results revealed PE prevents mitotic aberrations and GIN in NCM460 cells. They implied PE has the potential to protect human normal colon epithelial cells from genetic and mitotic damages. Coupled with our previous results that found PE elevates GIN in human colorectal cancer cells [8], one open issue is the molecular mechanisms underlying the dual actions of PE in modulating genomic integrity between normal and cancer cells. Once this question is answered, it will be valuable to develop the nutritional and clinical functions of PE in cells with different pathological states.

## Abbreviations

AMR	anaphase-to-metaphase ratio
BNC	binucleated cell
BubR1	budding uninhibited by benzimidazoles related 1
CB	chromatin bridge
CL	chromosome lagging
CMA	chromosome misalignment
GIN	genomic instability
Mad2	mitotic arrest deficient 2
MEF	mouse embryonic fibroblasts
MN	micronucleus
MNC	multinucleated cell
NB	nuclear bud
ND	nocodazole
NPB	nucleoplasmic bridge
PE	*Phyllanthus emblica*
SAC	spindle assembly checkpoint
